# Learning strengths from cultural differences: a comparative study of maternal health-related behaviors and infant care among Southern Asian immigrants and Taiwanese women

**DOI:** 10.1186/1472-698X-13-5

**Published:** 2013-01-22

**Authors:** Yen-Ching Chen, Shu-Hui Wei, Kuo-Wei Yeh, Mei-Yen Chen

**Affiliations:** 1 Out Patient Department, Chang Gung Memorial Hospital, Tunghwa North Road, Taipei, Taiwan; 2 Chang Gung Children’s Hospital, Tunghwa North Road, Taipei, Taiwan; 3 Chang Gung University of Science and Technology, 61363, Putz City, Chiayi County, Taiwan

**Keywords:** Immigrant, Infant health, Women's health, Lactation, Maternal-child health

## Abstract

**Background:**

Many studies have indicated that most immigrant women come from underdeveloped countries, and this can have negative effects on their lives, children’s adaptation to school, and medical care utilization. However, there is insufficient literature about differences in infant caretaking, pre-postpartum health care, and health outcome between immigrant and native Taiwanese populations. The aim of this study was to investigate the differences between Southern Asia immigrants and Taiwanese women in their access to medical care, postnatal growth, and infant care throughout the first six months postpartum.

**Methods:**

Comparative and descriptive designs were applied. Immigrant women were eligible if they visited three suburban settings of the Outpatient Department of Obstetrics and Gynecology and the Outpatient Department of Pediatrics in Northern Taiwan during the period up to six months postpartum.

**Results:**

Immigrant women appeared to have a lower frequency of antenatal examinations and obtained less health information from health care providers. However, they did not differ significantly from native Taiwanese women in maternal body size, postnatal growth curves, exclusive breastfeeding rates or vaccination awareness at the 6^th^ month postpartum.

**Conclusions:**

Learning strengths from cultural differences between immigrant and native women and closing the gaps in health inequality are important issues. Despite the limitation of small sample size, the present findings can be used as references to help health care providers to develop further health policies in Taiwan.

## Background

Female immigration due to cross-border marriage has gradually become more common in Taiwanese society. However, there is still insufficient literature about differences in infant caretaking, prepartum/postpartum health care, and health outcome between the immigrant and native Taiwanese populations. According to the Ministry of the Interior in Taiwan, up to the end of 2010 more than 386,000 families in Taiwan included a foreign or mainland Chinese spouse. Emigrants from mainland China, Hong Kong and Macau accounted for 65% of these families, with the remaining 35% involving spouses from other areas of the world [1]. Concerns have been raised about the impact of differences in lifestyle and culture on health during maternity and infant care [2]. Many studies have indicated that most immigrant women come from underdeveloped countries with backward economic conditions, so they are disadvantaged by race, social class and gender. These disadvantages can have negative effects on their lives, children’s adaptation to school, and medical care utilization [3-5].

According to a previous report, cultural differences become evident in cross-border marriages and relocations, causing disturbances in eating habits, interpersonal relationships and daily routines. Language barriers worsen social interactions, which include being unable to go out independently, to take public transportation, or to go shopping. They also cause difficulties in communicating with parents-in-law, which may give rise to further self-imposed isolation [6]. Immigrants who are affected may develop emotional symptoms of stress, anxiety, anger and regret if the situation persists [7,8]. Many physical disorders and illnesses can be attributed to the conditions described above and to social maladaptation. Symptoms of psychological disorders include headache, lost appetite, weight loss, and insomnia [9]. In addition, language barriers prevent effective communication with medical staff limit the immigrants’ use of healthcare services, and family members or friends become the major sources of information [7,10].

Some studies have demonstrated that new immigrant women with insufficient awareness and skills about breastfeeding have a low success rate in breastfeeding and a lack of knowledge of infant care related to safe bathing and vaccination. Lower rates of use of healthcare services such as antenatal examinations and cervical Pap smear tests have also been reported [3,11]. According to some studies on the promotion of infant growth [12,13], behavior can be positively affected by infant stimulation via body massage and by environmental change. Infant massage can also enhance the experience of parenthood, attachment, and positive interactions between parents and their infants.

Cultural adaptation and the demand for health care have been emphasized in immigrant-related studies [6,14]. However, comparative studies between immigrant and Taiwanese women concerning infant care, health outcome and stimulation of developmental growth are still lacking. Therefore, the purpose of this study was to compare immigrants and Taiwanese women with regard to antenatal healthcare use, maternal body weight and infant growth curves during the first six months postpartum.

## Methods

### Design and sample

Descriptive and comparative designs were applied in this study. Eligible subjects were immigrant women who were natives of Indonesia, Vietnam, and mainland China, and visited three suburban settings of the Outpatient Department of Obstetrics and Gynecology and the Outpatient Department of Pediatrics in Northern Taiwan between September 1, 2008 and August 31, 2010. Immigrant women were chosen by convenience samples. Taiwanese women selected immediately before or after the immigrant women from the same healthcare unit. A similar population size of Taiwanese women was selected as the comparison group. The inclusion criteria for immigrant women selected participants who: (1) were married to Taiwanese citizens and had emigrated no more than five years previously from their native countries; (2) were between six and eight months postpartum; (3) could communicate in Taiwanese and Mandarin; and (4) were willing to participate in the study. Exclusion criteria were: (1) premature labor and infant born with abnormality, (2) under six months postpartum, and (3) no regular check-up in the study setting. A sample size of 79 (total N=158) for each group would achieve 95% confidence in detecting an effect size of 0.45 using an independent t test with a significance level of 0.05. The actual sample size was 150; 11 were excluded because of incomplete data such as lack of records in the child health handbook.

### Procedure and Ethical considerations

This study was approved by the institutional review board of Chang Gung Memorial Hospital (No 96-1457A). The purposes of the study were explained to all invited participants and written informed consent was obtained from them prior to completion of the questionnaires. All participants were recruited and interviewed in one prenatal and one postnatal time point at a private room of the above three health institutions. Those participants who were unable to fill out the questionnaires provided the information to research assistants.

### Measures and data collection

(1) *Demographic characteristics of the mother*, including age, marital status, gestational history (e.g. delivery type, parity and gender), educational attainment, religion, height and body weight;

(2) *Process and outcome of infant care* was proprietarily prepared by the research team according to observations made in the clinic, a literature review, and the expert opinions of obstetricians and pediatricians. This information comprised breastfeeding, postnatal body weight, height, and head circumference within 1-6 months postpartum, types of stimulation used for infant development such as music, physical massage and environmental factors, and vaccination records and health-related information.

(3) *Infant health status*: Neonatal height, body weight, head circumference, and vaccination status were obtained from the records of the child health handbook.

The content validity of the above questionnaires was established through scoring by six content experts. A 5-point scale was used to assess content validity, where 1 indicated highly irrelevant or inappropriate content and 5 indicated highly relevant or appropriate content. The modified questions with scores of 4-5 points were categorized as appropriate content. The percentage appropriateness for each individual question was assessed by each specialist and a content validity index of 0.984 was calculated. In order to enhance the validity of measurement, the questionnaires were translated in advance into Vietnamese and Bahasa Indonesian by experts at a foreign language institution, enabling the immigrant women to understand them. Afterwards, five each of native Vietnamese and Indonesian women were invited to score the comprehensibility of the questionnaire and to provide feedback. The content of the questionnaires was adjusted accordingly and an appropriate version of the infant care status was finalized.

### Statistical analysis

Data were processed using SPSS software (version 17). An independent sample *t*-test was used to compare continuous variables between the immigrants and Taiwanese women, and a chi-square/Fisher exact test was applied to compare categorical data. A two-way mixed-factor ANOVA was used to examine neonatal body weight, head circumference and height at onset and at the first, second, fourth and sixth month after birth, with time as the repeated-measurement factor and ‘new immigrants’ and ‘native Taiwanese women’ being the between-subjects factors.

## Results

### Description of the participants

Of the 161 subjects selected, 150 completed the study. The average age of the immigrants was 28.3 years (SD=4.2), and more than half of them came from mainland China (Table 1). For the Taiwanese women, the average age was 33.5 years (SD=3.8). The average durations of marriage were 3.2 and 4.2 years, respectively. Most of the immigrants (66.7%) had graduated from junior high schools. More than half of them (61.3%) were non-religious. Nearly all of the immigrant participants (94.7%) were covered by the National Health Insurance (NHI) program, and most were unemployed (82.7%). Body mass index (BMI) had returned to the normal range by the 6^th^ month postpartum more frequently among the immigrants (77.3%) than the Taiwanese women. All of the Taiwanese participants had a minimum educational attainment of senior high school. Most of them had religious beliefs (64%) and were in employment (77.3%).

**Table 1:** Demographic characteristics between immigrant and Taiwanese women

**Variables**	**Immigrant**		**Taiwanese**		**t/χ**^**2**^	***p***
	***n***	***%***	***n***	***%***		
Age (year)	M=28.3	SD=4.2	M=33.5	SD=3.8	t= -7.92	<.001
Married year	M=3.2	SD=2.7	M=4.2	SD=3.1	t= -2.12	
Numbers of kids						.036
One	45	60.0	44	58.7	4.10	
Two	28	37.3	23	30.7		.129
Three and above	2	2.7	8	10.7		
Type of delivery^1^					26.06	
NSD	66	88.0	37	49.3		<.001
C/S	9	12.0	38	50.7		
Education^2^					a	
≦Secondary	50	66.7	0	0.0		
≧High school	25	33.3	75	100.0		<.001
Health insurance					a	
Yes	71	94.7	75	100		
No	4	5.3	0	0.0		
Occupation					54.15	0.12
Yes	13	17.3	58	77.3		
No	62	82.7	17	22.7		
Religion^2^					9.63	<.001
No	46	61.3	27	36.0		
Yes	29	38.7	48	64.0		
Regular antenatal check					a	
Yes	67	89.3	75	100.0		.002
No	8	10.7	0	0.0		
Mother participated class					12.18	
Yes	34	45.3	55	73.3		.006
No	41	51.7	20	26.7		
Spouse participated class					5.12	
Yes	18	24.0	31	41.3		<.001
No	57	76.0	44	58.7		
BMI at 6^th^ month ^3^						
Underweight (<18.5)	4	5.3	5	6.7	0.33	
Average (18.6~24.0)	58	77.3	55	73.3		.024
Overweight (>24.1)	13	17.3	15	20.0		
						.846

^1^ NSD: Normal spontaneously delivery, C/S: Cesarean section.

a Fisher’s exact test.

^2^Buddist, Daoism, Christian, Catholic.

^3^ BMI: body mass index (BW/ (height in meter square).

### Comparing maternal health-related behaviors

There were no statistically significant differences between the two groups in respect of parity, health insurance and exclusive breastfeeding rates during the first week, or in BMI values at the 6^th^ month postpartum (Table 1). The immigrant women differed significantly from the Taiwanese women in being younger (*t*=-7.92, p<0.01), not being as long married (*t*=-2.2, p<0.05), and having lower educational attainment (p<0.001) and a higher unemployment rate (p<0.001); also, fewer were covered by the NHI (p<0.05), and fewer were religious (χ^2^ =9.63, *p*<0.01). Taiwanese women showed a significantly higher percentage of cesarean sections (50.7%, χ^2^=26.06, *p*<0.001) and a higher frequency of antenatal examinations (100%, p<0.01). The frequency of attendance at learning programs for infant care was higher for Taiwanese women (χ^2^ =12.18, *p*<0.001) and their spouses (χ^2^ =5.12, *p*<0.05) than for immigrants.

### Comparing child care and infant growth

Table 2 shows that most of the participants in both groups (immigrant/native) were aware of vaccination-related information, including the time schedule (98.7%/97.3%), vaccination types (92%/94.7%), purpose (98.7%/100%), potential adverse effects (84%/93.3%), and management for adverse effects (83.8%/88%). A high percentage of immigrant women were unaware of information relating to new types of vaccines that were not covered by NHI reimbursement (p<0.001). Many immigrants (44%) obtained vaccination-related information from non-healthcare personnel (χ^2^ =7.68, *p*<0.01). The percentages of women in the two groups who were exclusively breastfeeding at the 6^th^ month postpartum were 46.7% and 32%, respectively. In terms of infant sleep, 72% of immigrants and 76% of Taiwanese women reported regular sleep patterns. However, maternal sleep disturbance relating to infant care was higher among Taiwanese women (58.7%, χ^2^ =14.36, *p*<0.001). Regarding stimulation for development while the infants were awake, Taiwanese women more frequently provided auditory stimuli such as music and talking (84%/68%, χ^2^ =5.26, *p*<0.05), physical massage (64%/46.7%, χ^2^ =4.56, *p*<0.05), or environmental stimulation such as outdoor activities (78.7%/29.3%, χ^2^ =36.74, *p*<0.001).

**Table 2:** Comparison of infant care between immigrant and Taiwanese women

**Variables**	**Immigrant**		**Taiwanese**		**p**
	**n**	**%**	**n**	**%**	
Vaccination time schedule^1^					1.000
Yes	74	98.7	73	97.3	
No	1	1.3	2	2.7	
Vaccination types^1^					.745
Yes	69	92.0	71	94.7	
No	6	8.0	4	5.3	
Purpose of vacination^1^					1.000
Very important	74	98.7	75	100	
Not important	1	1.3	0	0	
New type of vacine^1^					<.001
Yes	54	72.0	73	97.3	
No	21	28.0	2	2.7	
Receive health information^1^					.006
Health care providers	42	56.0	58	77.3	
Non health care providers	33	44.0	17	22.7	
Adverse effects of vacine^1^					.071
Yes	63	84.0	70	93.3	
No	12	16.0	5	6.7	
Manage the adverse effect					.460
Yes	62	83.8	66	88.0	
No	12	16.2	9	12.0	
Breastfeeding 1^st^ week					.683
Yes	61	81.3	59	78.7	
No	14	18.7	16	21.3	
Current breastfeeding					.150
Exclusive breastfeeding	35	46.7	24	32.0	
Full milk	20	26.7	29	38.7	
Mixed	20	26.7	22	29.3	
Sleep pattern of baby					.577
Regular	54	72.0	57	76.0	
Irregular	21	28.0	18	24.0	
Sleep disturbance of mother					<.001
No	54	72.0	31	41.3	
Yes	21	28.0	44	58.7	
Providing stimulation for baby					
Sound (e.g. music)					.022
Yes	51	68.0	63	84.0	
No	24	32.0	12	16.0	
Massage					.033
Yes	35	46.7	48	64.0	
No	40	53.3	27	36.0	
Environmental					<.001
Yes	22	29.3	59	78.7	
No	53	70.7	16	21.3	

^1^ Fisher’s exact test.

**Figure 1:**
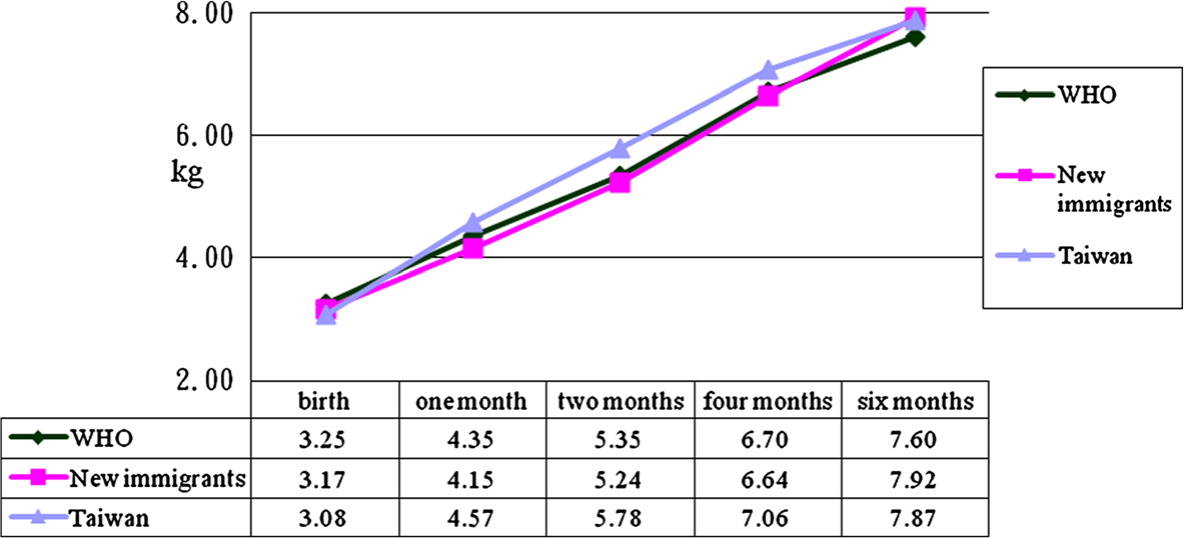
Differences of body weight among immigrant, Taiwanese women born infant and World Health Organization report.

**Figure 2:**
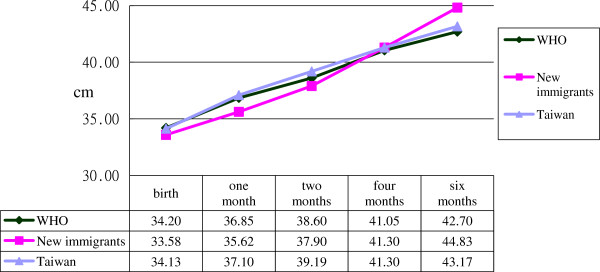
Differences of head circumference among immigrant, Taiwanese women born infant and World Health Organization report.

**Figure 3:**
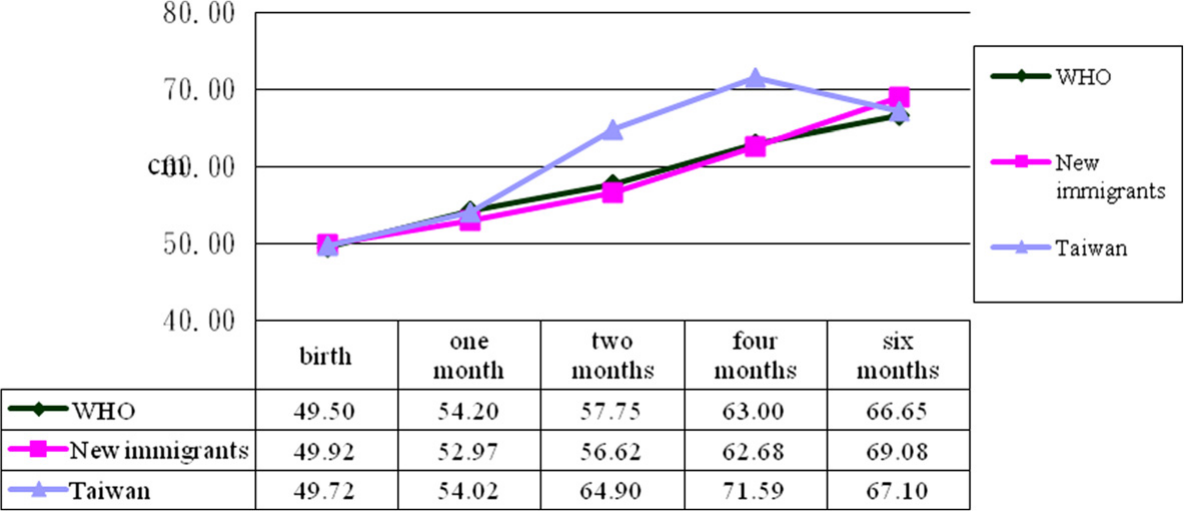
Differences of body height among immigrant, Taiwanese born baby and report from World Health Organization report.

Records of neonatal height, body weight and head circumference were compared with those published by the World Health Organization [15]. The results are depicted in Figures 1, 2 and 3, which show that infant height, body weight and head circumference were similar at the time of birth and during the first six months. Further analysis by two-way mixed-factor ANOVA showed no significant difference between the two groups of participants in neonatal height, body weight or head circumference of infants at birth or during the first six months postpartum (Table 3, p>0.05).

**Table 3:** Two-way mixed-factor ANOVA

**Health status**	**Immigrant Taiwanese mean**		**Mean square *F p***		
Body height (cm)	59.50	61.47	328.72	0.24	.626
Body weight (Kg)	5.49	5.68	2.88	1.18	.281
Head circumference (cm)	39.04	38.99	0.19	0.01	.919

## Discussion

To our knowledge, this is the first study comparing maternal health-related behaviors and infant care between immigrants and native women in Taiwan. The findings demonstrate the importance of respecting cultural differences and closing the gap between immigrants and native women concerning health inequality in maternal and child care.

### Respect for cultural differences

According to the current literature, immigrant women are relatively disadvantaged in economics, health status, quality of life, and the use of healthcare services [9,11]. However, in this study, no significant differences were found between the two groups in infant height, body weight or head circumference within six months postpartum. In addition, the average infant height, body weight and head circumference in both groups were very close to those recommended by the World Health Organization [15] for ideal infant development worldwide.

Obesity has been prevalent among postpartum women in many developed countries. In Taiwan, the importance of postpartum weight control has been highlighted by the Department of Health, Executive Yuan [16]. The ideal BMI for Taiwanese adults has been determined as 18.5-24; a BMI less than 18.5 indicates the individual is underweight and a BMI over 24 characterizes an overweight adult. These BMI values have been provided as guidelines for public health policies and have been included in health promotion programs. In this study, at the 6^th^ month postpartum, there was no significant difference between the BMIs of immigrants and Taiwanese women. In addition, both groups showed similar awareness of vaccination-related information such as time schedules, purpose, and the management of potential adverse effects of vaccination. Despite the small sample sizes in this study, the results could provide different ways of thinking about the health of immigrant women, especially for ways of respecting culture and assessing discrimination issues in Taiwanese society.

Many studies have recognized the benefits of exclusive breastfeeding for the health of mothers and infants. These benefits include the numerous immunological, psychological, social, economic and environmental advantages that breast milk provides; it is also the natural first food and ideal nutrition for the newborn [17]. However, this study found that exclusive breastfeeding among the immigrant women dropped from 81.3% in the first week after birth to 46.7% by the 6^th^ month postpartum. This result was consistent with a study conducted in Taipei city by Chen et al. [14], who found that the prevalence of exclusive breastfeeding at 6^th^ month postpartum among 210 immigrant mothers was 59%. Additionally, there were significant associated factors including breastfeeding experience among mothers-in-law, and the mainstream culture of Taiwan. Some studies have demonstrated that immigrant women with insufficient relevant awareness or skills are less efficient at breastfeeding [4,6]. According to the meta-analyses, breastfeeding promotion interventions increased breastfeeding rates at 4-6 weeks and at 6 months [17]. This suggests that providing individually-tailored breastfeeding-related counseling for immigrant women and their families is an important health strategy [18].

Significantly more cesarean sections (50.7%) were performed on Taiwanese women than immigrant women. According to the data published by the Taiwan National Health Insurance Bureau in 2010, the cesarean section rate was 38% in primary clinics and 35% in hospitals [19]. During the period 1992 to 2011, the average cesarean section rate in all clinical institutions was 35.5%. Concerns about non-natural birth have arisen from these high cesarean section rates among Taiwanese women. A previous study demonstrated that either poor sleep quality or insufficient physical activity during pregnancy was associated with a higher incidence of unplanned cesarean sections among Taiwanese women [20]. Some evidence indicates that increased cesarean section rates could be associated with high risk postpartum antibiotic treatment and severe maternal morbidity and mortality [17]. This finding is contrary to that of a study in England, where Fairley and Leyland [21] reported that caesarean section rates (17.4%) correlated with socioeconomic inequalities and area deprivation during the period 1980 to 2000: women in the most deprived areas were less likely to have an elective section than those in the most affluent areas. Although our study design did not assign patients randomly and the sample was small, the reason why Taiwanese women appear to have a high percentage of cesarean section deliveries merits exploration.

Our results showed that poor sleep quality related to sleep disturbance by children was higher among Taiwanese women. Furthermore, the exclusive breastfeeding rate decreased from 78.7% during the first week to 32% at the 6^th^ month postpartum in this group. Does employment, combined with poor sleep quality, cause the dramatic decrease in breastfeeding among Taiwanese women, since most of them must return to work two months after giving birth? Small sample sizes and a restricted sampling area could have limited the conclusiveness of these findings. Further studies should focus on the relationship between sleep quality and exclusive breastfeeding rate and the underlying reasons.

### Closing the gap concerning health inequality in maternal and child care

The immigrant women showed lower awareness than the Taiwanese women about the new types of vaccinations that are paid for out-of-pocket (28%), and less maternal (45.3%) and spouse-accompanied (24%) participation in learning programs for infant care. The major information sources for the immigrant group were family members and friends rather than medical staff. According to previous reports, this may be attributable to failure to acquire relevant information because of language barriers and less outdoor activity [6,7]. Therefore, the results suggest that medical staff need to improve the accuracy in delivering healthcare-related information to new immigrants, including ensuring that the content of education leaflets is comprehensible for them. In clinical facilities or primary healthcare centers, it would be beneficial to provide a translation platform or comparable resource for supporting attendees to overcome any language barrier.

Immigrant women also received fewer regular antenatal examinations (89.3%) and provided less stimulation (e.g. music, talking, outdoor activities) for infant development. These findings are consistent with other studies [22,23]. This is despite Taiwan’s National Health Insurance program, which was set up in March 1995 with the goal of providing high quality, affordable health care to all. In this study, 94.7% of the immigrant participants were covered by the program, but that was less than the 100% coverage for Taiwanese women. With regard to infant development, cognition can be promoted if multiple stimulations for development, such as language, sensory organ, sound, and ambient condition, are introduced at an early stage [12,13]. This study has demonstrated that immigrant women stimulate their infants’ sensory organs less frequently, particularly with physical environmental stimuli. Lower levels of educational attainment and lower economic strata may predispose the new immigrants to limited learning about infant-related stimuli from media such as toys, books and magazines. In Taiwan, studies involving stimuli for infant development are still lacking. However, from the perspective of infant care-related health promotion, professionals with related expertise should be assisting new immigrants by providing access to the knowledge and skills for infant-related sensory stimuli.

## Limitations

This study has some limitations. First, the data are cross sectional so they can only describe associations between socioeconomic factors and health-related behaviors. Second, the selection of participants was restricted to the local health care centers in Northern Taiwan within the first six months postpartum. Further, our findings might not be generalizable to other immigrant groups in Taiwan, since the baseline demographic criteria including educational attainment, the rate of NHI coverage, and employment status were not reciprocal between the two groups, so the conclusions that can be drawn from the results are limited.

## Conclusion

Although the study has some limitations, it had strengths in demonstrating that there was no significant difference between the two groups in mean infant body height, body weight, or head circumference and those obtained from the WHO. Furthermore, there were no significant differences between new immigrants and Taiwanese women in postpartum body size, sleep disturbance, exclusive breastfeeding rate, vaccinations, and management of post-vaccination adverse effects. A high percentage of Taiwanese infants were delivered by caesarean section and the women complained of insufficient sleep. Immigrant women received health information from non-healthcare providers; they also received fewer antenatal examinations and less spouse-accompanied participation in learning programs for infant care, and provided less stimulation for infant development. Future studies are required that will incorporate a comparative approach for different populations with larger sample sizes into the study design.

## Competing interests

The authors declare that they have no competing interests.

## Authors' contributions

YCC and MYC designed the study and drafted the manuscript. SHW collected, analyzed and interpreted the data and drafted the manuscript. KWY conceived of and participated in the design of this study and interpreted the data. All authors read and approved the final manuscript.

## Pre-publication history

The pre-publication history for this paper can be accessed here:

http://www.biomedcentral.com/1472-698X/13/5/prepub

## References

[B1] Ministry of Interior Immigrants Agency, MOI Foreign spouse population and Mainland (including Hong Kong and Macao) spouse population Retrieved on 2010 from http://iff.immigration.gov.tw/mp.asp?mp=2

[B2] Bhutta ZA Hasan BS Haws RA Community-based interventions for improving perinatal and neonatal health outcomes in developing countries: A review of the evidence Pediatrics 2005 115 2 519 617 15866863 10.1542/peds.2004-1441

[B3] Guendelman S Schauffler HH Pearl M Unfriendly shores: How immigrant children fare in the U.S. health system Health Aff 2001 20 1 257 265 10.1377/hlthaff.20.1.257 11194849

[B4] Lin CC Chen SH The effects of nursing guidance on the breastfeeding patterns, knowledge and attitudes of foreign spouses Evid Based Nurs 2007 3 2 161 169

[B5] Wang HH Yang YM The health of Southeast Asian women in transnational marriages in Taiwan Hu Li Za Zhi 2002 49 2 35 41

[B6] Chen MJ Tang CS Jeng HM Chiu AWH The maternal and child healthcare needs of new immigrants in Taipei J Nurs Res 2008 16 4 307 319 10.1097/01.JNR.0000387318.50880.b5 19061177

[B7] Huang YC Mathers NJ Postnatal depression and the experience of South Asian marriage migrant women in Taiwan: Survey and semi-structured interview study Int J Nurs Stud 2008 45 6 924 931 10.1016/j.ijnurstu.2007.02.006 17418193

[B8] Kuo WH Wilson TE Holman S Fuentes-Afflick E O’Sullivan MJ Minkoff H Depressive symptoms in the immediate postpartum period among Hispanic women in three U.S. cities J Immigr Health 2004 6 4 145 153 16228697 10.1023/B:JOIH.0000045252.10412.fa

[B9] Yang YM Wang HH Cross-cultural comparisons of health-related quality of life between Taiwanese women and transnational marriage Vietnamese women in Taiwan J Nurs Res 2011 19 1 44 52 10.1097/JNR.0b013e31820bebcd 21350386

[B10] MacFarlane A Glynn L Mosinkie P Murphy A Responses to language barriers in consultations with refugees and asylum seekers: A telephone survey of Irish general practitioners BMC Fam Pract 2008 9 8 10.1186/1471-2296-9-8 19102735 PMC2637872

[B11] Lee LC Yin TJC Yu S Prenatal examination utilization and its determinants for immigrant women in Taiwan: An exploratory study J Nurs Res 2009 17 1 73 81 10.1097/JNR.0b013e3181999ee8 19352231

[B12] Bradley RH Caldwell BM The HOME inventory and family demographics Dev Psychol 1984 20 2 315 320

[B13] Chen MY Hsu CC Preliminary evaluation of reliability and validity of Home Observation for Measurement of Environment and Infant Scales of Development in Taiwan Hu Li Za Zhi 1991 38 4 119 128

[B14] Chen TL Tai CJ Chu YR Han KC Lin KC Chien LY Cultural factors and social support related to breastfeeding among immigrant mothers in Taipei City, Taiwan J Hum Lact 2011 27 1 41 48 10.1177/0890334410376519 20858846

[B15] World Health Organization, WHO The WHO child growth standards *R*etrieved October 5, 2011from http://www.who.int/childgrowth/en/

[B16] Department of Health, Executive Yuan, Taiwan Our country children will grow the diagram of curves to use the World Health Organization newest children to grow the standard Retrieved on 2011from http://www.nhi.gov.tw

[B17] Imdad A Yakoob MY Bhutta ZA Effect of breastfeeding promotion interventions on breastfeeding rates, with special focus on developing countries BMC Public Health 2011 11 S24 10.1186/1471-2458-11-S3-S24 21501442 PMC3231898

[B18] Digirolamo A Intention or experience? Predictors of continued breastfeeding Health Educ Behav 2005 32 2 208 226 10.1177/1090198104271971 15749967

[B19] National Health Insurance, NHI Statistical Annual Reports Retrieved on 2012 from http://www.nhi.gov.tw/english/index.aspx

[B20] Tsai MS Huang CM Kuo WM Wu HM Lee MY Physical activity, sleep quality and unplanned cesarean section in pregnant women J Nurs Health Sci 2010 6 1 13 23

[B21] Fairley L Leyland AH The influence of both individual and area based socioeconomic status on temporal trends in caesarean sections in Scotland 1980-2000 BMC Public Health 2011 11 330 10.1186/1471-2458-11-330 21592328 PMC3114726

[B22] Calderon-Larranaga A Gimeno-Feliu LA Macipe-Costa R Poblador-Plou B Bordonaba-Bosque D Prados-Torres A Primary care utilization patterns among an urban immigrant population in the Spanish National Health System BMC Public Health 2011 11 432 10.1186/1471-2458-11-432 21645335 PMC3128023

[B23] Lin ML Wang HH Prenatal examination behavior of Southeast Asian pregnant women in Taiwan: A questionnaire survey Int J Nurs Stud 2008 45 5 697 705 10.1016/j.ijnurstu.2006.12.005 17339036

